# The relationship between free fatty acids and mitochondrial oxidative stress damage to trophoblast cell in preeclampsia

**DOI:** 10.1186/s12884-022-04623-0

**Published:** 2022-03-31

**Authors:** Lingling Jiang, Jianying Yan

**Affiliations:** Fujian Maternity and Child Health Hospital, College of Clinical Medicine for Obstetrics & Gynecology and Pediatrics, Fujian Medical University, 18 Daoshan Rd, Fuzhou, Fujian, 350001 China

**Keywords:** Preeclampsia, Oxidative stress, Free fatty acids, Trophoblast cells, Mitochondrial

## Abstract

**Aim:**

To investigate the effects of free fatty acids on mitochondrial oxidative stress and the pathogenesis of preeclampsia.

**Methods:**

Human primary trophoblast cells at 6–8 weeks of gestation were retrieved and cultured to 70–80% confluence, then incubated in serum from women with a normal pregnancy (normal pregnancy group), women with preeclampsia (PE group), and a combination of serum from women with 24 h preeclampsia-like symptoms and free fatty acids (FFA group). Mitochondrial membrane potential was assessed by fluorescent dye concurrent with detection of membrane channel conversion pore activity by fluorescence microscope. Enzyme labeling instruments and RT-PCR were used to detect mitochondrial DNA (mtDNA) levels.

**Results:**

The preeclampsia and free fatty acids groups both exhibited significantly higher levels of mitochondria oxidative stress damage when compared to the normal pregnancy group. However, no significant differences in mitochondrial oxidative stress damage were observed between the FFA and PE groups.

**Conclusions:**

Serum free fatty acids might play an important role in the pathogenesis of preeclampsia by enhancing mitochondrial oxidative stress damage.

## Introduction

Preeclampsia is a pregnancy-related hypertensive disorder that is the leading cause of maternal and neonatal mortality. It is one of the main causes of intra-uterine growth retardation, affecting 3–8% of all pregnant women and accounting for 10–15% of all perinatal mortality [1]. Although preeclampsia has been extensively studied, the underlying pathogenesis remains unclear. Oxidative stress damage and lipid metabolism disorders are associated with preeclampsia. Oxidative stress is the imbalance between oxidation and anti-oxidation, that is the production of reactive oxygen species (ROS) exceeds the oxidative defense ability of the body. Placental mitochondria are important components of oxidative stress injury. Free fatty acids (FFAs) are those fatty acids in serum that are not esterified with glycerol and cholesterol. They are produced primarily by the lipolysis of subcutaneous and visceral fat. Compared with other lipids, FFA react earlier and more sensitively to lipid metabolism disorders. Previous studies have found a significant relationship between increased serum FFA concentrations and preeclampsia pathogenesis [2, 3]; however, the specific mechanism remains unclear. The presence of FFAs can promote ROS production and aggravate oxidative stress damage. This study investigated the participation of FFA in the pathogenesis of preeclampsia and its role in the increase of oxidative stress damage in trophoblast cells.

## Materials and Methods

### Villi tissues

All subjects were admitted to the same hospital over a period from October 2017 to September 2018 and were between 20–32 years in age. All pregnancies were from patients who had elected voluntarily termination at 6–8 weeks of gestation and no patients demonstrated any surgical or pregnancy-related complications. Sterile villi were obtained from all consenting patients by vacuum aspiration, which were then used to inoculate subsequent trophoblast cell cultures.

### Specimen preparation

Venous blood (3 mL) was collected from a peripheral vein before initiating treatment (within 24 h of admission). The serum was obtained by centrifugation at 3,000 rpm for 15 min and then incubated in a 56℃ water bath for 30 min for complement inactivation. The sample was then sterilized using a microporous filter, followed by addition to a Nutrient Mixture F-12 (DMEM/F12) culture medium at a final concentration of 10%.Relevant literature was reviewed for preeclampsia diagnostic criteria [4]. The general characteristics of all subjects are provided in Table 1. Patients with pregnancy-, surgical-, or internal medicine-related complications were excluded from this study. All the subjects had a singleton pregnancy.

**Table 1:** The clinically relevant indicators of pregnant women

group	case	Age (years)	gestational age (weeks)	pre- pregnancy BMI (Kg/m^2^)	pregnancy BMI (Kg/m^2^)		Blood pressure	
						SP	DP	MAP
PE group Normal group	30 30	27.4 ± 3.3 27.3 ± 3.0	37.2 ± 2.0 37.4 ± 1.6	19.9 ± 1.2 19.7 ± 0.8	25.5 ± 1.2 25.9 ± 1.9	159.4 ± 5.5* 113.6 ± 6.3	97.6 ± 3.2* 72.1 ± 7.0	118.2 ± 2.5* 86.0 ± 5.6

(Compared with the normal group, * *P* < 0.05)

### FFA preparation

The stock solution (5 mmol/L) was prepared by dissolving fatty acids in ethanol, which was then added to DMEM/F12 medium (Invitrogen, Carlsbad, CA, USA). The final working solution was obtained by diluting the above mixture to its final desired concentration [5].

### Cell culture

A previously reported digestive method was used to culture first-trimester human trophoblast cells [6]. The decidua in the villous tissue was completely detached and repeatedly washed with PBS to effectively remove blood clots and impurities. The intermediate shaft section was resected, and the vill tissues were cut into 1-mm^3^ pieces before storage in centrifuge tubes. A mixture of 0.25% trypsin, 0.1% collagenase, and deionized water was added to the tube and incubated for digestion in a 37 °C water bath for 25 min. DMEM/F12 containing 10% fetal bovine serum (FBS) was added after digestion. The suspension then filtered through a 200-mesh stainless steel screen, then centrifuged at 3000 rpm for 5 min and the resulting supernatant discarded. Cells were inoculated into a culture incubator at 37 °C with a 5% CO_2_ atmosphere after suspension in DMEM/F12 (with 10% FBS). The remaining cells were inoculated for 2 h to remove fibroblasts by differential adherent culture.

Trophoblast cells were identified by immunocytochemistrystaining. Most of the cells were positive for cytokeratin (CK7) and negative for vimentin (Vimentin), indicating a high level of purity for trophoblasts. Cells were sealed in tubes for diaminobenzidine staining. Trophoblast cells were also identified using aStrep Avidin–Biotin Complex Kit (American RD). All procedures were performed according to the manufacturer’s instructions. Four percent paraformaldehyde was used to fix the trophoblast cells, 0.5% Triton was used to disrupt cell membranes, and 5% Bovine Serum Albumin was used for protein blocking. Samples were then incubated with the primary antibodies against cytokeratin 7 or vimentin, followed by the secondary antibody sheep anti-mouse IgG. After diaminobenzidine coloration and hematoxylin re-staining, alcohol was used for gradual dehydration. Trophoblastcells were then sealed in transparent xylene and photographed under a microscope.

### Cell groups

Cells were cultured in DMEM/F12 medium containing 10% of the serum collected from patients with normal pregnancy (normal pregnancy group), patients with preeclampsia (PE group), and 10% of a serum obtained by combining serum from women who had 24 h of preeclampsia-like symptoms and free fatty acids (FFA group). Cells and supernatant from the three groups were separately collected after a 24 h incubation.

### Measuring mitochondrial oxidative stress damage

#### Mitochondrial membrane potential measurement

Mitochondrial membrane potential was measured using amitochondrial membrane potential assay kit (JC-1) (American AAT Bioquest Inc). An aliquot containing 5 × 10^4^ mL^−1^viable trophoblast cells was incubated in DMEM/F12with 10% FBS in 6-well culture plates. Cells were later divided into groups upon achieving 80–90% growth and cultured in different serums for 24 h. Carbonyl cyanide 3-chlorophenylhydrazone (CCCP, 20 µL) was added into a neutral well 20 min prior to the assay and served as the positive control. Plates were first washed with PBS, followed by 1 mL of JC-1 working solution and 1 mL of medium in each well.The plates were incubated at 37 °C in a 5% CO_2_ incubator for 20 min, then washed two times with 4 °C 1 × JC-1 buffer. Finally, 2 mL of DMEM/F12 containing 10% FBS was added to each plate. The relative ratio of red and green fluorescence was observed under a fluorescence microscope.

#### Measurement of mitochondrial permeability transition pore activity

Aliquots containing 5 × 10^4^ mL^−1^of viable trophoblast cells were used to inoculate 6-well culture plates, then divided into groups and cultured with different serums for 24 h after achieving 80–90% growth. All procedures were performed according to the operating instructions of the mitochondrial permeability transition pore (MPTP) fluorescence detection kits (American Genmed Scientifics). The RFU value was detected using a fluorescence enzyme labeling instrument. In addition, the MPTP pore activity was calculated six times at 5-min intervals.

#### Mitochondrial DNA expression

A genomic DNA extraction kit (from American RD Company and provided by JieMei Ltd) was used to extract and analyze genomic DNA, according to the manufacturer’s instructions. The probes and primers were designed using Express software version 2.0 (Applied Biosystems, Foster City, CA), and the sequences were obtained from the relevant literature [7]. The Human Mitochondrial DNA Revised Cambridge Reference Sequence was used to obtain the mitochondrial DNA primer sequences. Primers to amplify the sequences (264 bp sequence length) were as follows: 5'-ACG ACC TCG ATG TTG AAT C-3' for the upstream primer and 5'-GCT CTG CCA TCT TAA CAA ACC-3' for the downstream primer. The probe sequence was 5'-FAM-TTC AGA CCG GAG TAA TCC AGG TCG-TAMRA-3'. The reference gene was 28S rRNA with a fragment length of 102 bp; forward primer 5'-TTA AGG TAG CCA AAT GCC TCG-3', reverse primer 5'-CCT TGG CTG TGG TTT CGC T-3', gene sequence 5'-FAM-TGA ACG AGA TTC CCA CCT GTC CCT-ACC TAC TAT C-TAMRA-3. The parameters of the PCR cycle were as follows: 2 min denaturation at 50 °C, 10 min activation of the AmpliTaq Gold DNA polymerase at 95 °C, and 40 cycles of 10 s at 95 °C, and 40 s at 60 °C. The target gene was quantified by the 2^−△△ct^ method on the premise that the amplification efficiency of the target gene was identical to amplification of the internal reference gene.

### Statistical analysis

Statistical Package for Social Science Software (SPSS17.0) was used for data analysis. Data are expressed as mean ± SD. The difference between the two groups was compared by t-test. Multiple groups comparison was made using ANOVA. A *P*-value < 0.05 was considered statistically significant.

## Results

### General characteristics

The gestational and maternal ages, the pregnancy body mass index, the pre-pregnancy body mass index, the diastolic pressure, the mean arterial pressure, and the systolic pressure of normal pregnant women and severe preeclampsia women were listed in Table 1.

### Trophoblast cell identification

**Fig. 1:**
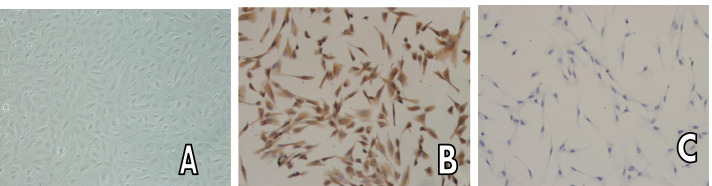
primary culture trophoblast cells and identification A. Normal group, B. PE group, C. FFA group

Trophoblast cells were examined for morphology using an inverted microscope, revealing epithelial-like cells that had formed a 1-cell thick layer and contained cells rich in cytoplasm and showing irregular polygonal lines with large, oval nuclei (Fig. 1A). Immunocytochemical detection indicated 95% cell purity, with positive detection of CK7 in the cytoplasm (brown, negative in Fig. 1B) and negative detection of Vimentin (Fig. 1C).

### Mitochondrial membrane potential measurement

**Fig. 2:**
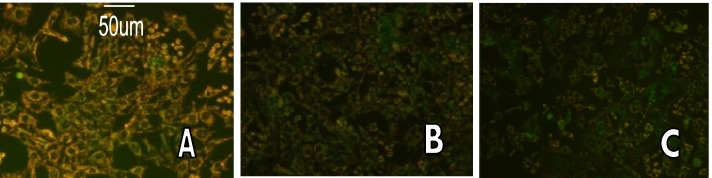
Fluorescence microscopy detected the mitochondrial membrane potential (Bar = 50 μm, magnification × 100. A. Normal group, B. PE group, C. FFA group

The normal group cells had a higher amount of orange-red fluorescence in comparison to the PE and FFA groups. Most trophoblast cells in the PE and FFA groups fluoresced green, indicating transmembrane potential depolarization and, therefore, a decrease in mitochondrial membrane potential and morphological changes (Fig. 2). The average depolarization ratio of mitochondrial membrane potential was 1.13 ± 0.58% in the normal group, 64.27 ± 2.27% in the PE group, and 63.23 ± 1.99% in the FFA group. The amount of mitochondrial membrane potential depolarization in the normal group was significantly lower than in the PE and FFA groups (P < 0.05). No significant differences in mitochondrial membrane potential depolarization were observed between the PE and the FFA groups (Fig. 2).

### Assessment of mitochondrial permeability transition pore activity

**Fig. 3:**
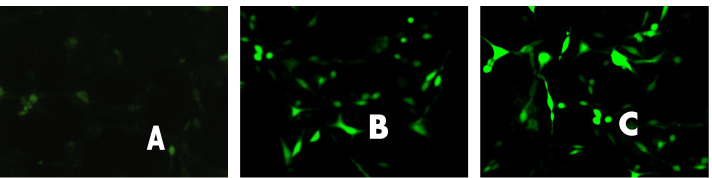
Fluorescence microscopy detected the activity of the mitochondrial permeability transition pore. Normal group PE group FFA group mean

The normal group had a weaker green fluorescence signal, which was even weaker within the mitochondria. The corresponding quenching rate was very low, indicating few openings of mitochondrial membrane channel pores. The PE and FFA groups had higher green fluorescence intensity compared to the normal group, suggesting the presence of a greater number of mitochondrial membrane channel pore openings (Fig. 3).

### mtDNA expression

**Fig. 4:**
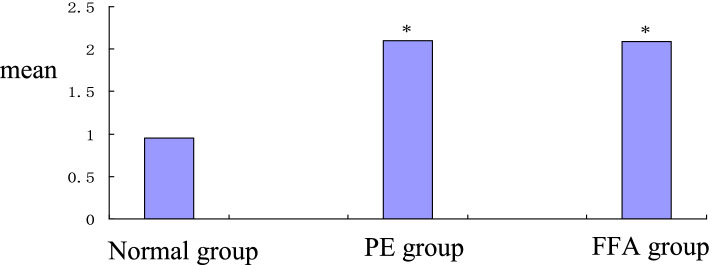
Comparison of the relative mtDNA expression of each group *P < 0.05

The results of the expression of mtDNA for the groups are shown in Table 2. The mean relative mtDNA expression in the normal group (0.95 ± 0.02) was significantly lower than in the PE (2.10 ± 0.03) and FFA (2.09 ± 0.07) groups. However, there was no significant difference in the relative expression of mtDNA between the PE and FFA groups (Fig. 4).

**Table 2:** The expression of mtDNA for each group

group	28S rRNAC_T_	mtDNAC_T_	mtDNA/28S rRNA
Normal group	16.64 ± 0.31	16.57 ± 0.28	0.95 ± 0.02
PE group	15.79 ± 0.09	16.86 ± 0.08	2.10 ± 0.03*
FFA group	15.84 ± 0.10	16.91 ± 0.14	2.09 ± 0.07*

(Compared with the normal group, * *P* < 0.05)

## Discussion

### Preeclampsia and disturbance of lipid metabolism

Preeclampsia is a major obstetric complication and the leading cause of maternal and perinatal mortality [8]. Preeclampsia has a multi-factorial etiology, and the specific pathogenesis remains unclear. Many studies have confirmed that a lipid metabolic disorder is one of the clinical symptoms of preeclampsia and could potentially directly participate in its pathogenesis [9, 10].

Standard fetal growth requires the placental transport of fatty acids. Consequently, lipid levels in maternal serum significantly increase to provide for fetal growth and development, especially during middle and late pregnancy. These changes are accompanied by increased protein production-as underlying lipid metabolism is altered during pregnancy-typically in proportion to gestational development. However, a prior study [11] indicated that preeclampsia patients have higher blood levels of FFA as compared to those with a normal pregnancy, suggesting that these patients may have abnormalities in gestational lipid metabolism. FFAs are active metabolic lipids that are especially useful in the early diagnosis of dyslipidemia due to their higher sensitivity, as compared to apolipoproteins, triglyceride, high-density fatty acids, and low-density fatty acids [12].

Many studies have observed higher FFA levels in patients with preeclampsia, suggesting that elevated serum FFAs might constitute a predisposing factor for preeclampsia [13, 14]. Yan et al. [15] observed that both increased serum FFA levels and placental mitochondrial oxidative stress injuries might be two leading factors in the pathogenesis of preeclampsia. Furthermore, increased serum FFA levels can enhance placental mitochondrial oxidative stress damage in pregnant women. However, there is currently no clear link between FFA-mediated mitochondrial oxidative stress damage and the pathogenesis of preeclampsia.

### Preeclampsia and mitochondrial oxidative stress damage

Oxidative stress, through increased levels of ROS, can cause significant damage by tipping the balance between the production of antioxidants and prooxidants, as several studies have suggested that oxidative stress plays a significant role in the pathogenesis of preeclampsia [15, 16]. Interestingly, placental mitochondria are the leading source of oxidative stress during pregnancy and are also commonly the targets of oxidative stress damage [17]. Therefore, excessive ROS can result in mitochondrial damage, resulting in functional and structural changes to trophoblast cells, and potentially causing placental ischemia and hypoxia, ultimately playing an important role in the pathogenesis of preeclampsia.

A recent report observed that ROS can induce the opening of MPTPs via a redox-sensitive switch on the conversion pore [18]. Therefore, opening of trophoblastic MPTPs might result in increased in mitochondrial permeability, mitochondrial depolarization, and a subsequent decrease in/disappearance of membrane potential. Furthermore, decreased membrane potential promotes MPTP opening, which can cause a positive feedback loop. Previously, women with preeclampsia were observed to have a greater abundance of mtDNA expression and oxidative stress damage in placental mitochondria, while also demonstrating a decreased mitochondrial membrane potential in trophoblast cells [3].

### FFA and mitochondrial oxidative stress damage

Goglia F et al. [19]  observed that FFA might cause mitochondrial dysfunction by increasing oxidative stress in cells, while Robinson et al. [2] revealed diminished mitochondrial membrane potential levels in umbilical vein endothelial cells when cultured using the serum of women with preeclampsia. These cells had higher FFA membrane potentials in normal pregnant women when compared to their preeclampsia-like counterparts. In addition, the serum FFA was determined to cause damaging effects of mitochondria in endothelial cells. In this study, we showed that the mitochondrial oxidative stress damage in the FFA group was significantly higher than in the normal group, while there were no significant differences between the FFA and the PE groups. This suggests that FFAs most likely take part in the pathophysiology of preeclampsia by increasing the oxidative stress damage of mitochondria. 

## Conclusion

This study demonstrated that FFAs can play an active role in the pathophysiology of preeclampsia by increasing trophoblastic mitochondrial oxidative stress damage. Additionally, FFA was confirmed to lower mitochondrial membrane potential, while also increasing both mitochondrial permeability and mtDNA expression. These findings shed new light on the pathogenesis of preeclampsia and might provide a new direction for its prevention. Further studies are needed to investigate the interactions between lipid metabolism and oxidative stress injury, which will provide significant clinical insights into the pathogenesis of preeclampsia, thereby potentially revealing new treatment avenues.

## Data Availability

The datasets used and analyzed during the current study available from the corresponding author on reasonable request.
